# Supplementary material for: Can Chemotherapy Negatively Affect the Specific Antibody Response toward Core Vaccines in Canine Cancer Patients?
Source: Vet Sci. 2023 Apr 20;10(4):303. doi: 10.3390/vetsci10040303 (PMC10143758; doi:10.3390/vetsci10040303)
Supplement: Supplementary file 1 [file vetsci-10-00303-s001.zip › vetsci-2325399-supplementary.pdf]

Table S1 VacciCheck: correspondence between S scale units and antibody titers, sensitivity, and specificity for Canine Parvovirus type 2 (CPV-2), Canine Distemper Virus (CDV), and Canine Adenovirus type 1 (CAdV-1)

	**CPV-2 (%)**	**CDV (%)**	**CAdV-1 (%)**
S0	<1:20	<1:8	<1:4
S1	1:20	1:8	1:4
S2	1:40	1:16	1:8
**S3 (threshold)**	**1:80**	**1:32**	**1:16**
S4	1:160	1:64	1:32
S5	1:320	1:128	1:64
S6	1:640	1:256	1:128
>S6	>1:640	>1:256	>1:128
Sensitivity (%)	*88*	*100*	*94*
Specificity (%)	*100*	*92*	*93*
